# Literary Notes

**Published:** 1903-03

**Authors:** 


### LITERARY NOTES.

THE *Internal Secretions and the Principles of Medicine*, is the title of a new work by Dr. Charles E. de M. Sajous, of Philadelphia, the first volume of which is announced to appear the latter part of February, 1903. Dr. Sajous is so well known to the profession of medicine over the whole world through the "Annual of the Universal Medical Sciences" and its successor the "Analytical Cyclopedia of Practical Medicine," that a work from his pen will be received with favor. The study of the ductless glands is becoming one of vast interest, and may lead to a new era in practical medicine. From the advanced sheets of the preface and summary of contents of the first volume, we are led to believe that this work will be of absorbing interest to the student of internal medicine. It is published by the F. A. Davis Company, of Philadelphia.

THE Annual Report of the Department of Health of the City of Niagara Falls for 1902 has been issued. It is a well prepared document, showing in detail the conditions affecting the health of the city, its vital statistics, and many data of importance relating to public sanitation. It reflects the highest credit upon Dr. C. G. Leo-Wolf, the efficient health officer. During the year there were 412 marriages in that city, of which 231 were of non-resident couples. The traditional belief that Niagara Falls is a city of honeymoons seems to be well founded.

THE *Journal of Tuberculosis*, the well-known quarterly magazine devoted to the consideration of the prevention and treatment of tuberculosis, began its fifth volume in January, 1903. This number contains six original communications by prominent writers, domestic and foreign, besides translations, reviews of current literature, and editorial comment. The journal is edited by Drs. Karl and Silvio von Ruck, of Asheville, N.C., and merits the attention of all persons interested in the problems of which it treats.

*Northwest Medicine* is the title of a new journal that has recently appeared and is published at Seattle, Washington. The first number bears the date of January, 1903, and it presents a good front. It is edited by Dr. Clarence A. Smith and is published by the Washington Medical Library Association.

*Modern Medical Preparations* is the title of a handy little brochure, issued by Victor Koechl & Co., of New York, which gives important information concerning the products of the Farbwerke, formerly Meister Lucius and Brüning, Hoechst-on-Main, and vereinigte Chemische Werke, formerly Benno Jaffé

and Darmstadter, Charlottenburg, Germany. It gives the clinical history, therapeutics, bibliography, and price of each article, and is a most useful book of reference.

---

The New York State Hospital for the care of Crippled and Deformed Children, located at Tarrytown, has issued its second annual report, which is for the year ending September 30, 1902. During this period thirty-five patients have been treated, of whom ten have been discharged. About 150 applications for admission have been made during the year, but owing to the restricted accommodations the larger portion could not be accepted. If the hospital had been equipped with 500 beds, Dr. Shaffer, the surgeon-in-chief, says he could have filled them in a month with deserving patients. Let the state enlarge the hospital that its good work may continue and its benefit extend to the needy crippled and deformed children.

---

Prof. John Uri Lloyd's famous satires, the first of which, "The Mother of Sam Hill's Wife's Sister," was published in the September *Criterion* (1901), are resumed in the January number with the fourth paper of the series, "Sam Hill, Sheriff of Knowlton, Kaintuck," and purport to be related by "Chinnie Bill Smith," the famous story teller of "Stringtown On The Pike." These satires, written exclusively for the *Criterion*, will be illustrated by Martin Justice, whose character studies are second to none in the magazine field. Prof. Lloyd's inimitable style and daring, yet kindly humor, will be a rare treat to *Criterion* readers. A deeper meaning will be read between the lines of these unusual papers by thoughtful minds. The next paper, "Why a Kentuckian Stands with his Back to the Stove, The Testing of Milinda," by Sam Hill, will appear in the March *Criterion*, and the remaining stories during the year 1903.

